# General anesthesia for cesarean section in a pregnant woman with systemic vascular malformation: a case report

**DOI:** 10.1186/s40981-023-00682-0

**Published:** 2023-12-14

**Authors:** Noriko Takeuchi, Misa Koshihara, Akira Motoyasu, Joho Tokumine, Harumasa Nakazawa, Mine Ozaki, Tomoko Yorozu

**Affiliations:** 1 Department of Anesthesiology, Kyorin University School of Medicine, 6-20-2 Sinkawa, Mitaka, Tokyo, 181-8611 Japan; 2 Department of Plastic and Reconstructive Surgery, Kyorin University School of Medicine, 6-20-2 Sinkawa, Mitaka, Tokyo, 181-8611 Japan

**Keywords:** Vascular malformation, Cesarean section, Difficult airway

## Abstract

**Background:**

Vascular malformations are composed of morphologically abnormal vascular tissue, and when located in the head and neck region, they can make it difficult to secure the airway during general anesthesia.

**Case presentation:**

A 28-year-old pregnant woman with vascular malformations in the pharynx was scheduled to undergo a cesarean section, for which spinal anesthesia was initially chosen. However, after magnetic resonance imaging results revealed the presence of multiple vascular malformations in the lumbar multifidus muscles, spinal anesthesia was considered to be of high risk. Thus, the patient was subjected to general anesthesia tracheal intubation under sedation, and the course of the surgery was without complications.

**Conclusions:**

Because the pathophysiology and clinical sequelae of vascular malformations may be involved in complications, thorough presurgical evaluation of the patient’s physical condition and careful anesthesia planning should be done.

## Background

Vascular malformations are comprised of dysplastic vessels lined by normal endothelium and are caused by abnormal morphogenesis of the vascular tissue [1]. Therefore, vascular malformations differ from neoplasms such as hemangiomas [1]. Vascular malformations are often observed in the head and neck region and can cause airway stenosis and/or obstruction [2, 3]. Furthermore, they make it difficult to secure the airway during general anesthesia [4–6]. Vascular malformations sometimes occur in the limbs, trunk, brain, spinal cord, and uterus [1]. We herein present our experience of general anesthesia for cesarean section in a pregnant woman with systemically present vascular malformations. Written consent for publication was obtained from the patient to report the case.

## Case presentation

A 28-year-old female was scheduled for a cesarean section at 37 weeks of pregnancy due to a growing vascular malformation in the perineum with possible bleeding during vaginal delivery. She had vascular malformation in the neck and extremities since birth, with a diagnosis of venous malformation. She underwent surgical removal of vascular malformation of the dorsal foot and temporal head in infancy, had sclerotherapy in the pharynx, and resection of that in the left hand and neck under general anesthesia with tracheal intubation under sedation at 26 years of age. No other comorbidities in found in her medical history and preoperative laboratory data were within normal limits. She was receiving aspirin 100 mg daily as an antithrombotic and analgesic, which was switched to low-molecular weight heparin 75 U/kg/h 1 week before surgery. Recently, she was not able to sleep in the supine position due to breathlessness and always slept in the right decubitus position.

**Fig. 1:**
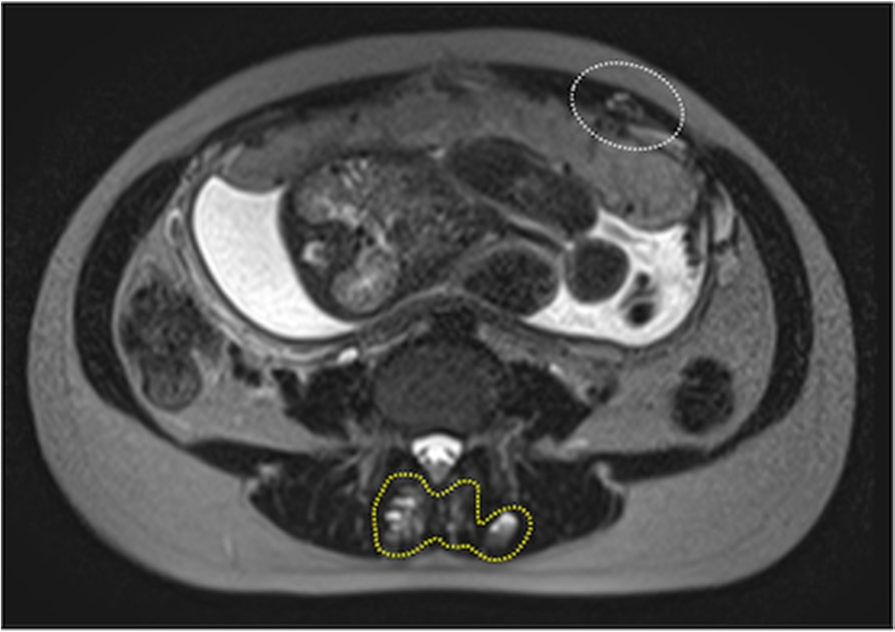
Transverse view of MRI between the third and fourth lumbar vertebrae. The MRI T2-weighted image shows multiple vascular malformations in the lumbar multifidus muscles (circled by the yellow dashed line), and the lateral edge of the left rectus abdominis muscle (circled by the white dashed line)

**Fig. 2:**
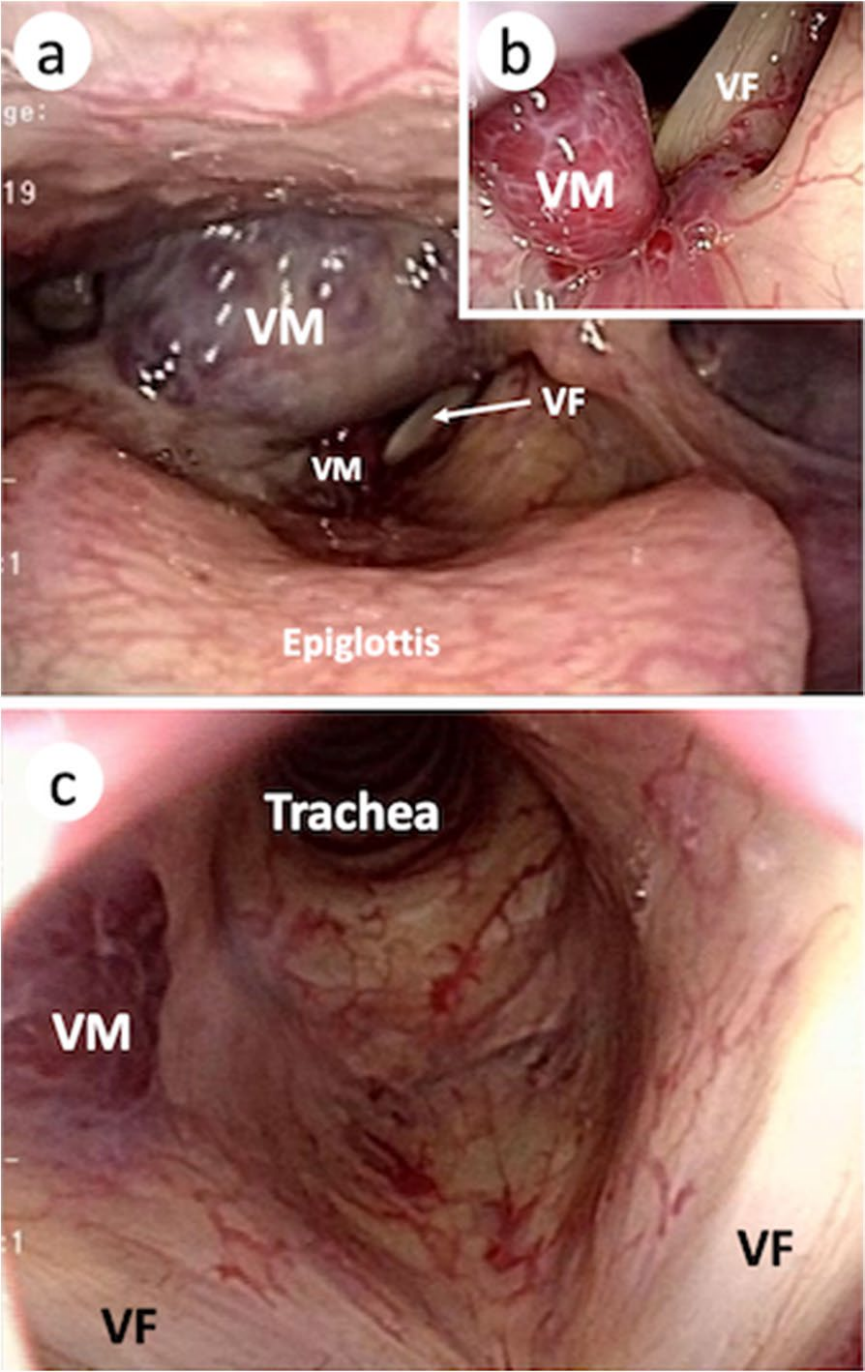
Vascular malformations in the glottis. **a** Overview of the larynx. **b** Vascular malformation on the right side of the glottis. **c** Vascular malformation in the subglottic region. VM, vascular malformation; VF, vocal fold

Although spinal anesthesia was initially planned, an MRI examination to rule out vascular abnormalities in the uterus revealed new multiple vascular malformations in the lumbar multifidus muscles, but not in the epidural space (Fig. 1). Fiberscopic examination revealed two vascular malformations in the right glottis (Fig. 2a, b), which enlarged after surgery 2 years ago along with another vascular malformation in the subglottic region (Fig. 1c).

After a discussion with anesthesiologists, obstetrician–gynecologists, radiologists, and plastic surgeons, another general anesthesia was planned to be induced after fiberoptic intubation under sedation. In the case of the impossibility of securing the airway during anesthesia, namely, “cannot intubate and cannot oxygenate,” an emergency front-of-neck access was scheduled as a backup plan. However, the otolaryngologist determined that an emergency tracheotomy could not be implemented due to a residual vascular malformation in the neck. The anesthesiologists determined via ultrasound that needle cricothyroidotomy was possible, although vascular malformation was present nearby. Therefore, the anesthesiologists chose needle cricothyroidotomy for emergency front-of-neck access, and spinal anesthesia was considered as an alternative.

On the day of surgery, the patient’s vital signs were checked, and high-flow nasal oxygenation was carried out after entering the operating room. The location of the cricothyroid membrane was marked on the skin during ultrasonography. Fentanyl 50 μg was administered intravenously, and a target-controlled infusion of propofol was initiated. Topical anesthesia with an 8% lidocaine spray was administered with five puffs at each side of the pharynx. Then, high-flow nasal oxygenation was administered at a speed of 10 L/min and then gradually increased to 70 L/min. While observing the patient’s state of consciousness and bispectral index (BIS), the target concentration gradually increased to 1.0 μg/mL and was controlled for BIS 85, aiming for Ramsay Sedation Scale 3. Fentanyl was also administered intermittently at a dose of 50 μg, reaching up to a total amount of 150 μg. The above procedures were performed slowly over 30 min. Topical anesthesia of the glottis was performed using 2% lidocaine 2 mL through the suction channel of a flexible bronchoscope. After 5 min, awake fiberoptic intubation was induced. Bronchoscopy progressed to the glottis while avoiding the application of extra force against the vascular malformation. Then, 2 mL of 2% lidocaine was sprayed again onto the tracheal mucosa through the glottis. The patient did not complain of pain, discomfort, or gag reflex, and the fiberscope was passed through the glottis without resistance. Furthermore, 2 mL of 2% lidocaine was applied to the tracheal mucosa. A well-lubricated reinforced tracheal tube with an inner diameter of 6 mm was positioned with a fiberscope guide.

After confirming the tracheal tube placement, the target concentration of propofol was increased to 3.0 μg/mL, remifentanil was started at 0.2 μg/kg/min, and then rocuronium 50 mg was administered. Subsequently, a cesarean section was performed. The baby was born asleep and had an Apgar score of 2 at 1 min that increased to 7 at 5 min after manipulations. The surgery was completed without complications. The operative time was 51 min, with a blood loss of 871 mL and an in–out balance of 659 mL.

Postoperatively, while maintaining anesthesia, the patient was observed using flexible bronchoscopy in the supine and semi-sitting positions to check for bleeding from the enlarged vascular malformations. No change in the size of vascular malformation and bleeding occurred, and extubation was performed smoothly. The total time for anesthesia was 118 min. Intravenous fentanyl infusion was performed for postoperative analgesia. Peripheral nerve blocks for postoperative analgesia were not considered due to vascular malformations in the abdominal wall (Fig. 1). The postoperative course was uneventful, and 1 week later, the patient and the baby were discharged.

## Discussion

Depending on the embryological origin, vascular malformations are mainly classified into arteriovenous and fistulae, venous, capillary, and lymphatic [1]. Combined forms of them are often found in the same patient [1]. When vascular malformation occurs at a very early stage of embryological development, it has proliferative properties similar to those of an angioblast [1]. This means that they can have a high recurrence rate after treatment, and proliferation can also be provoked by trauma, surgery, and hormones (e.g., pregnancy) [1, 7].

The patient in this report had a mixed type of vascular malformation, which was mainly diagnosed as venous malformation. In addition, the size of vascular malformations in the pharynx increased with pregnancy. Diep et al. reported a case of airway stenosis during pregnancy caused by increased oral arteriovenous malformation [5]. Furthermore, Ghosh et al. described a case of sudden hemorrhage and difficulty in securing the airway in a pregnant woman with large lingual arteriovenous malformation, in which spinal anesthesia was induced after successful awake intubation using a nasal fiberscope [6]. In their case, the increased arteriovenous malformation gradually returned to its previous size after the delivery. In the present case, the vascular malformation of the pharynx became larger during pregnancy, and that of the lumbar back was noticed incidentally. Surgery in pregnant women with vascular malformation requires a thorough physical examination and detailed imaging before anesthesia.

The influence of pregnancy on the size of vascular malformations in the pharynx is an important issue. Duyka et al. reported that progesterone receptors were identified in vascular malformation [8]. However, Kulungowski et al. reported that the progesterone receptor was not expressed in the vascular malformation [9]. The conflicting results may be attributed to the diversity of hormone sensitivity in each vascular malformation. It is known that certain vascular malformations may be increased by pregnancy [10]. Apart from hormonal effects, the increased circulating blood volume associated with pregnancy may increase the size of venous malformations. The vascular malformations in the pharynx are rare [11]; hence, few cases of pharynx vascular malformations have been reported in pregnant women. For this reason, the effect of pregnancy on vascular malformation in the pharynx cannot be predicted.

Howton et al. reported airway obstruction following tracheal extubation after resection of intraoral vascular abnormality, which required emergency tracheostomy [4]. In this case study, the tracheal tube was removed without complications. Avoiding overhydration and the semi-sitting position during extubation may have effectively prevented an increase in the size of the venous malformations.

Vascular malformation can present with a variety of pathologic signs and clinical sequelae. Hence, a detailed preoperative examination and meticulous anesthesia planning should be done for a patient with vascular malformations.

## Data Availability

Not applicable.

## Abbreviations

MRI: Magnetic resonance imaging
BIS: Bispectral index
